# Yogurt fortified with omega‐3 using nanoemulsion containing flaxseed oil: Investigation of physicochemical properties

**DOI:** 10.1002/fsn3.2571

**Published:** 2021-09-06

**Authors:** Khadijeh Almasi, Seyedeh Sara Esnaashari, Masood Khosravani, Mahdi Adabi

**Affiliations:** ^1^ Department of Medical Nanotechnology School of Advanced Technologies in Medicine Tehran University of Medical Sciences Tehran Iran; ^2^ Department of Medical Nanotechnology Faculty of Advanced Sciences and Technology Tehran Medical Sciences Islamic Azad University Tehran Iran

**Keywords:** flaxseed oil, nanoemulsion, omega‐3, yogurt

## Abstract

Flaxseed oil as a natural ingredient has many health benefits due to the rich contents of omega‐3 fatty acids. However, its use in food formulations is limited because of low aqueous solubility, easy oxidation owing to the unsaturated nature of the fatty acids such as omega‐3. The aim of this study was to prepare a stable nanoemulsion containing flaxseed oil and investigate the fortification of yogurt with this nanoemulsion compared with fortification with bulk flaxseed oil. The nanoemulsion of flaxseed oil‐in‐water was obtained by low‐energy emulsification method. Optimized nanoemulsion contains 3% (w/w) flaxseed vegetable oil, 36% (w/w) surfactant, 10% (w/w) co‐surfactant, and 51% (w/w) deionized water as a continuous phase. The result of transmission electron microscopy (TEM) showed that the optimal size was about 60 nm, which was stayed stable for 11 months. The results of gas chromatography (GC) indicated that the amount of omega‐3 in nanoemulsion containing flaxseed oil was 27.3% and 19.8% after 7 days and 11 months, respectively. The turbidity results indicated the transparency of nanoemulsion after 11 months as well. The results of centrifuge experiments and thermal stress cycles exhibited that the optimized nanoemulsion was physically stable without any sign of creaming, phase separation, and cracking. In addition, pH and acidity of the yogurt fortified with nanoemulsion containing flaxseed oil were 4.22 and 1.41 wt%, respectively. In conclusion, fortifying yogurt with the nanoemulsion containing flaxseed oil can be considered as a solution to increase solubility, bioavailability, and protection of omega‐3.

## Graphical abstract

Omega‐3 is an essential nutrient for human. But, due to highly unsaturated nature, they are readily oxidized and result in unpleasant taste and smell. Nanoemulsion technology has been considered as an efficient method for omega‐3 protection. Therefore, flaxseed oil as a herbal source of omega‐3 fatty acid was loaded into oil‐in‐water nanoemulsion via the low‐energy emulsification method. Nanoemulsion containing flaxseed oil was used to fortify yogurt, which can be a proper functional food due to its high protein and calcium content.

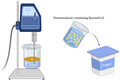

## 1 INTRODUCTION

Omega‐3 fatty acids belong to the family of unsaturated fatty acids (PUFAs). They are essential not only for their positive effects on the heart, brain, eyes, joints, and skin, but also for the human condition and behavior (Connor, 2000; Qin et al., 2017). Omega‐3 fatty acids play an important role in the prevention of coronary heart disease, high blood pressure, diabetes, arthritis, other inflammatory and autoimmune disorders, and cancer (Ajith & Jayakumar, 2019; Tur et al., 2012). Many studies encourage adequate consumption of omega‐3 fatty acids for pregnant and lactating women, in order to support the overall health of the fetus and the complete retinal and brain development (Connor, 2000; Helland et al., 2003; Ruxton et al., 2007; Von Schacky, 2020). The first member of the omega‐3 family is alpha‐linolenic acid (ALA, 18: 3n‐3), which is not synthesized by the human body. On the other hand, using omega‐3 supplements in capsule form is common only among a small population and its bioavailability may be minimized due to the incomplete absorption and degradation by the digestive system. According to these reasons and its important physiological roles in the human body, omega‐3 is therefore essential in the diet. The required level of omega‐3 fatty acids in the diet can be achieved by using a variety of foods fortified with omega‐3. Although several foods fortified with omega‐3 are available on the market, there are technical challenges in their production, transportation, storage, bioavailability, and sensory acceptance (Kolanowski & Berger, 1999; Walker et al., 2015). Utilization of omega‐3 oils as a potential nutrient is limited because of their physical and chemical properties. Due to their highly unsaturated nature, they are susceptible to oxidation to easily produce hydro peroxides and consequently unpleasant taste and smell, which are undesirable for the consumers. To overcome this problem, using nanoemulsion technology has been considered as an efficient solution (Bush et al., 2019; Klinkesorn et al., 2005). When the emulsion has a uniform distribution of particles with median size of below 100 nm, it is considered as a nanoemulsion which its properties including transparency, colloidal stability, and the surface to volume ratio are improved compared with conventional emulsions; (Amin & Das, 2019; Kentish et al., 2008). Nanoemulsions can be used as an efficient technique for fortifying food products with omega‐3 because they can protect the oil from oxidation, mask unwanted taste, and increase oral bioavailability (Huang et al., 2010). In a study by Alfaro et al. (2015), a frozen yogurt was fortified with a nanoemulsion containing purple rice bran oil with fat droplets size range of 150–300 nm and the effect of the nanoemulsion on the physicochemical characteristics of frozen yogurt was determined. In another research by Zhong et al. (2018), physicochemical properties of yogurt fortified with fish oil/γ‐Oryzanol nanoemulsion were investigated. The results demonstrated a significant reduction in the acidity and syneresis, a decrease in peroxide value, and higher retention of omega‐3 contents in the yogurt fortified with the nanoemulsion compared with yogurt with fish oil/γ‐oryzanol. Gharehcheshmeh et al. (2020) studied the qualitative properties of fortified yogurt produced with a sweet almond and sesame oil nanoemulsion using Span 80 and Tween 80 as an emulsifier. The results indicated that pH and syneresis reduced using the incorporation of sweet almond and sesame oil nanoemulsion containing 0.5% emulsifier, while the acidity, malondialdehyde formation, and antioxidant activity increased. Overall, sesame oil with 0.25% of the emulsifier was suggested for the production of fortified yogurt. Despite the fact that nanoemulsions utilization for the fortification in food products is increasing, there is still a necessity to optimize and formulate nanoemulsions containing omega‐3 which keep the taste, shelf life, and other physical properties of the food product.

Consumption of omega‐3 plant sources is essential for vegetarians and pregnant women who are prohibited from eating fish. In this study, flaxseed oil, a herbal source of alpha‐linolenic acid (about 55%), was used as a dispersed phase of oil‐in‐water nanoemulsion. The amount of omega‐3 in nanoemulsion containing flaxseed oil, the stability, and the transparency of nanoemulsion were investigated. In addition, pH and acidity of yogurt fortified with nanoemulsion containing flaxseed oil were compared with control yogurt and yogurt fortified with flaxseed oil. Yogurt because of high protein and calcium contents is often placed in healthy food list which can be used for the development of various health‐promoting functional foods. Therefore, in this study, nanoemulsion containing flaxseed oil was fortified into yogurt as a healthy food which is still a major factor affecting consumer preference.

## 2 MATERIALS AND METHODS

### 2.1 Materials

Flaxseed oil was purchased from Barij Essence pharmaceutical company. Fatty acid composition of flaxseed oil is seen in table 1. Tween 80, Span 80, and ethanol were supplied from Merck chemicals (Germany). Commercial yogurt purchased from local supermarket was from Kalleh dairy company.

**TABLE 1:** Fatty acid composition of flaxseed oil

Fatty acid	Structure	Flaxseed oil (g /100 g)
Palmitic acid	C16:0	6.14
Stearic acid	C18:0	4.73
Oleic acid	C18:1n−9	18.36
Linoleic acid	C18:2n−6	15.98
Linolenic acid	C18:3n−3	53.56

### 2.2 Methods

#### Preparation of nanoemulsion

Low‐energy method was used for the preparation of nanoemulsions containing flaxseed oil. Surfactants were selected by HLB method. Tween 80 and Span 80 were used as non‐ionic surfactants. In order to find out the best ratio of Span 80 to Tween 80, the HLB value of the surfactant mixture was screened in the range of 11–15. Flaxseed oil with a HLB of 3.23 and deionized water was considered as dispersed phase and continuous phase, respectively. Ethanol (98%) was used as co‐surfactant to increase the stability of the nanoemulsion. To obtain the optimal formulation, samples were prepared with different ratios of materials. To prepare each sample, the oil and hydrophobic emulsifier (Span 80) were first weighed and mixed using a heating magnetic stirrer (Heidolph, Germany) at 500 rpm for 10 min at room temperature. Subsequently, Tween 80 was added as a hydrophilic emulsifier, and after 10 min, ethanol was added dropwise to the resulting mixture. At the end of the process, while the mixture was still stirring by the magnet, deionized water was added dropwise. One hour after mixing, changes in color, transparency, and integrity of the mixture were visually analyzed. The stability of stable samples was monitored regarding the formation of no sediment, creaming, two‐phase separation and also no changes in nanoparticle size within one month. Unstable samples were removed from the screening process, and finally, the most stable sample with an optimized formulation was chosen for characterization tests.

#### Stability tests

The nanoemulsion containing flaxseed oil with an optimal formula was subjected to three different thermodynamic stability tests. These accelerated storage tests were performed using centrifuge and thermal stress tests including freeze–thaw and heating–cooling cycles, in order to evaluate the physical stability of the nanoemulsion. Nanoemulsion was centrifuged at 3200 *g* for 30 min at room temperature for 3 cycles. For freeze–thaw assessment, nanoemulsion was stored in deep freezer (at −20℃) for 24 h. Then, it was kept at room temperature. This cycle was repeated for 3 times. The heating–cooling cycles were performed 3 times at 4℃ and 40℃ for at least 48 h kept in each temperature. After each stage, the nanoemulsion was visually analyzed for transparency, sign of phase separation, creaming, and sedimentation. The droplet size in each step was also measured by the dynamic light scattering (DLS) method (Scatteroscope, K‐One, Korea). The long‐term stability of optimal nanoemulsion containing flaxseed oil was also studied. After 11 months of storage in glass container at room temperature (25℃), in the refrigerator (4℃) and incubator (45℃), all above‐mentioned experiments were performed (Ghiasi et al., 2019).

#### Characterization of optimized nanoemulsion

##### Droplet size and size distribution

Dynamic light scattering method was applied to determine the size of the droplets and their distribution. All of the samples were diluted with deionized water with 1:3 ratio, placed in the cuvette, and gently shaken to be mixed thoroughly. All measurements were made at room temperature, and the median particle size (d50) was obtained (*n* = 3).

##### Morphology and structure

The structure, shape, and the size of nanoemulsion particles with optimal formulation were also examined by transmission electron microscopy (TEM) (LEO 906, Zeiss, Germany) operating at 100 KV. To prepare the sample, one drop of nanoemulsion containing flaxseed oil was poured on a carbon‐coated grid and incubated at room temperature for 1 h. Then the extra amount was removed with a filter paper. After drying at room temperature, the sample was characterized.

##### pH

Inolab Multi 9,420 (WTW‐Germany) was used to measure pH. First, the pH meter was calibrated with a buffer and then the pH of each sample was measured for three times at room temperature and the fixed number was read.

##### Turbidity

The turbidity of optimal nanoemulsion containing flaxseed oil was evaluated at the first day and after 11 months of storage at room temperature through measuring the absorbance at wavelength of 600 nm by a UV‐Visible spectrophotometer (Cecil CE 7,250, England).

#### Determination of omega‐3 in nanoemulsion containing flaxseed oil

The amount of omega‐3, which is the main component of flaxseed oil, was investigated by GC 7890B (Agilent, USA). In order to investigate the level of omega‐3 in the nanoemulsion containing flaxseed oil, it was necessary to separate the oil and aqueous phases of the nanoemulsion. In other words, nanoemulsion should be destabilized for analyzing the extracted oil by GC. Direct analysis of fatty acids by GC is difficult, especially if unsaturated fatty acids are considered. Therefore, it is better to form fatty acid esters, because normally fatty acid methyl esters (FAMEs) are suitable for GC. The method of performing each steps of this test is as follows:

##### Separation of the oil phase from the aqueous phase

In order to separate the two phases of the nanoemulsion, physical instability method was used. According to the Institute of Standards and Industrial Research of Iran (ISIRI) 8,818 protocol, 100 mg of nanoemulsion was dissolved in 2 ml of normal hexane solvent and 1 ml of methanol was added. The mixture was placed on a magnetic stirrer for 10 min and then sonicated by a FAPAN 150 UT ultrasonic homogenizer for 10 min (the sample was placed inside a cold water cooling jacket to prevent overheating). In the next step, the sample was centrifuged at room temperature and a clear liquid supernatant was extracted.

##### Preparation of fatty acid methyl esters

There are several methods for preparing fatty acid methyl esters. In this study, by rapid internal methylation under catalyst‐alkali conditions according to ISIRI 13126–2 protocol, methyl esters were prepared using trans methylation with methanolic potassium hydroxide.

##### Analysis of omega‐3 profile by GC

Gas chromatography was used to analyze the level of omega‐3. 1 µl of sample was injected into the device, the injector temperature was 250℃, and the detector temperature was 270℃. The temperature of the oven at 50℃ was kept unchanged for 1 min; then, it reached 198℃ gradually and remained constant for 55 min. The formed fatty acid methyl esters were separated on the highly polar stationary phase according to their chain length, saturation or unsaturation state, and spatial position of the double bonds.

#### Preparation of fortified yogurt

Kalleh Icelandic yogurt containing 9% protein and 0% fat was purchased from supermarket and selected as a simple yogurt and control sample (Y), since non‐fat or low‐fat yogurts are popular due to their nutritional and potential therapeutic characteristics. The yogurt sample containing bulk flaxseed oil (YB) was prepared by adding 150 mg of flaxseed oil, containing approximately 75 mg of omega‐3 to 100 g of simple yogurt and fully mixed by stirring. Besides, the optimal nanoformulation with exactly the same amount of flaxseed oil was also added to the control yogurt to obtain the yogurt fortified sample with nanoemulsion (YN). The physical and chemical properties of these three samples were characterized and compared with each other at two time points (first and 21st day of storage in refrigerator according to the expiration date of yogurt).

#### Characterization of yogurt

##### pH

The pH was measured using inolab Multi 9,420 pH meter (WTW‐Germany) and 2,852 ISIRI method. The pH meter calibrated with two buffers with pH of 4 and 7. Subsequently, 10 grams of the sample was weighed and placed in 50 ml beaker. The pre‐calibrated pH meter electrode was positioned inside the beaker. Electrode and sample were kept in contact for at least 45 s. The pH of the sample was read.

##### Acidity

Acidity of yogurt samples was measured according to ISIRI 2,852 method. The yogurt was gently stirred using heating magnet stirrer (Heidolph, Germany), until it was completely uniform; then, 9 or 18 grams of the yogurt was weighed and poured into a suitable beaker and same amount of deionized water was added to it. In the next step, 0.5 ml of phenolphthalein reagent as indicator was added to sample and titrated with 0.1 N sodium hydroxide. This process continued until the solution color turned into pink, which lasted for at least 5 s. Acidity was calculated according to the percentage of lactic acid using the following formula.

0.009 g of lactic acid is equivalent to 1 ml of consumed sodium hydroxide 0.1 N.

$$\text{Acidity}\;\text{percentage}=\frac{\left(N\times 0.009\times 100\right)}{V}$$

where in: *N* = The ml of consumed sodium hydroxide 0.1 N.

V = sample Volume.

## 3 RESULTS AND DISCUSSION

### 3.1 The preparation of vegetarian omega‐3 nanoemulsion systems

Selection of the right hydrophilic and lipophilic surfactants and their appropriate ratio is necessary for nanoemulsion preparation by low‐energy method. In this regard, the HLB (Hydrophilic–Lipophilic Balance) method was used; Span 80 and Tween 80 were selected as hydrophobic and hydrophilic surfactant, respectively. Span 80 with HLB value of 4.3 and Tween 80 with HLB value of 15 were categorized as water‐in‐oil (W/O) and oil‐in‐water (O/W) emulsifiers. To determine the best ratio of Span 80 to Tween 80, samples containing 3% oil (w/w), 10% (w/w) co‐surfactant, and a total of 30% (w/w) surfactant with the HLB value of 11–15 were screened. All of the samples were visually analyzed. The most stable nanoemulsion of flaxseed oil with the smallest droplet size was observed with the HLB value of 12.91 (Table 2). In addition, the total amount of surfactants was also investigated (Table 3). The most stable sample, which was clear, was attained with 36% surfactant. Finally, the optimal nanoemulsion formulation contains 3% (w/w) flaxseed oil, 28.97% (w/w) Tween 80, 7.03% (w/w) Span 80 and 10% (w/w) ethanol, and 51% (w/w) deionized water.

**TABLE 2:** The preparation of emulsion samples with HLB values in the range of 11–15 and the different amounts of surfactant mixtures and the fixed amount of total surfactants

Sample	Span 80	Tween 80	Total surfactants	HLB
1	11.21	18.79	30	11
2	9.81	20.19	30	11.5
3	8.97	21.03	30	11.8
4	7.75	22.25	30	12.3
5	7.01	22.99	30	12.5
6	6.17	23.83	30	12.8
7	5.05	24.95	30	13.2
8	4.21	25.79	30	13.5
9	3.36	26.64	30	13.8
10	2.8	27.2	30	14
11	1.4	28.6	30	14.5
12	0	30	30	15
13	6.37	23.63	30	12.6
14	6.45	23.55	30	12.7
15	6.17	23.83	30	12.8
16	5.89	24.11	30	12.9
17	5.61	24.39	30	13
18	5.97	24.03	30	12.87
19	5.94	24.06	30	12.88
20	5.92	24.08	30	12.89
21	5.89	24.11	30	12.9
22	5.86	24.14	30	12.91
23	5.83	24.17	30	12.92

**TABLE 3:** The preparation of emulsion samples with different amounts of surfactant mixtures and total surfactants

Sample	Span 80	Tween 80	Total surfactants
24	5.97	24.03	30
25	6.06	24.94	31
26	6.25	25.75	32
27	6.45	26.55	33
28	6.64	27.36	34
29	6.84	28.16	35
30	7.03	28.97	36
31	7.23	29.77	37

The amount of flaxseed oil loaded with this method was about 3%, which is due to the highly hydrophobic feature of flaxseed oil. As a result, a large amount of surfactant is required to prepare a stable O/W nanoemulsion. Tadros et al. (2004) also claimed that low‐energy method for nanoemulsion preparation needs high amount of surfactants.

### 3.2 Physicochemical characterization of flaxseed oil nanoemulsion

**FIGURE 1:**
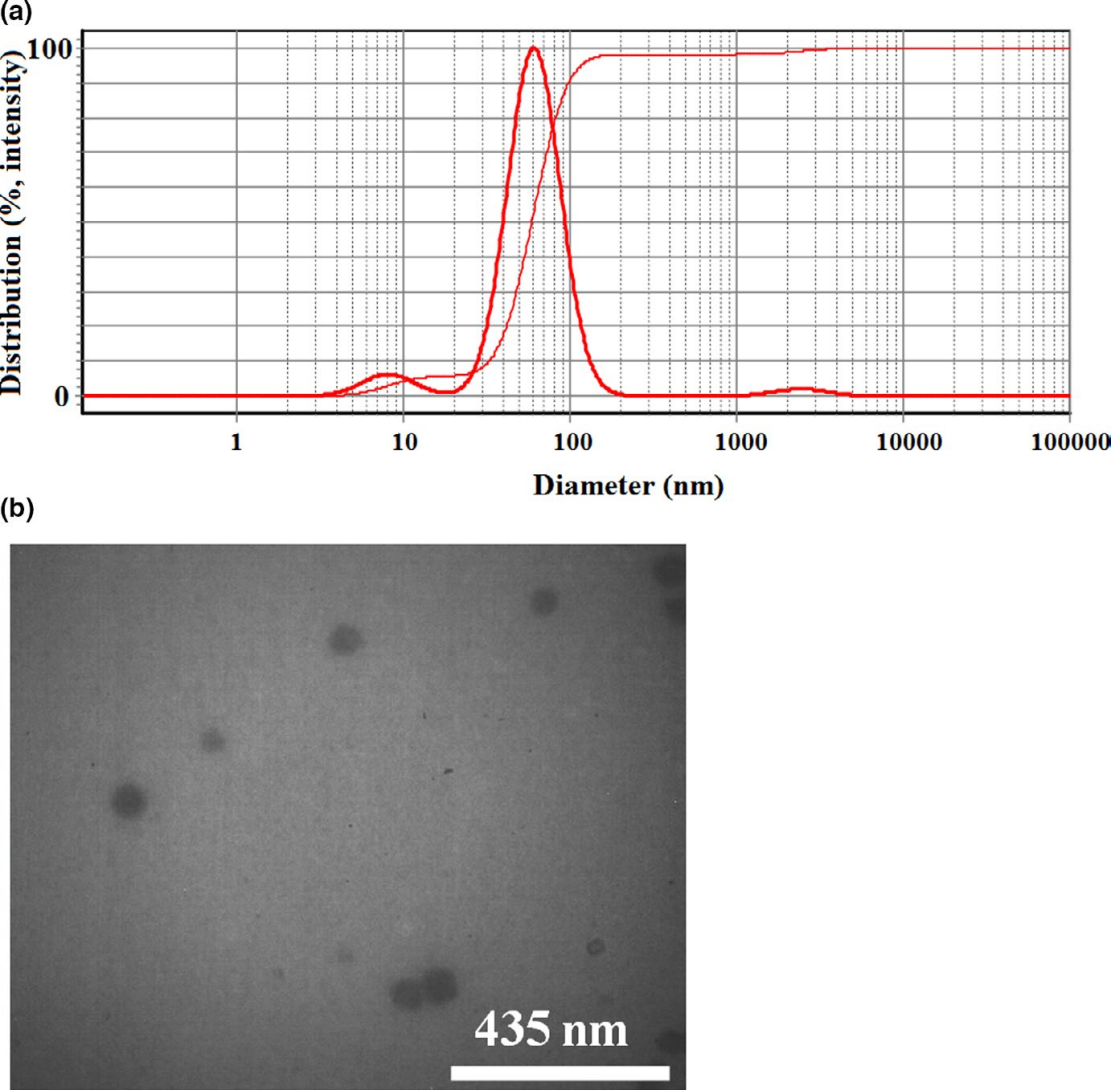
Dynamic light scattering of the optimized nanoemulsion containing flaxseed oil (a), and TEM image from nanoemulsion containing flaxseed oil (b)

The median droplet size of the optimal nanoemulsion formulations was about 60 nm according to the DLS measurements (Figure 1a). The results of TEM images showed spherical particles (Figure 1b). In addition, the size of particles in TEM was well‐consistent with DLS results.

The results of both centrifuge experiments and thermal stress cycles (freezing–thawing and heating–cooling) indicated that the optimized nanoemulsion was physically stable without any sign of creaming, phase separation, and cracking. Also, long‐term examination of the samples revealed that the samples remained stable for 48 h and maintained its physical stability for several months. A similar result was reported by Carpenter and Saharan (2017) that unstable emulsions were separated within 24 h, while stable emulsions never experienced phase separation for 90 days. In the present study, the nanoemulsion prepared with the optimal formulation was stable for up to 11 months and no sign of instability was observed.

The pH value of optimized flaxseed oil nanoemulsion was 6.24, which is acceptable for the oral consumptions.

**FIGURE 2:**
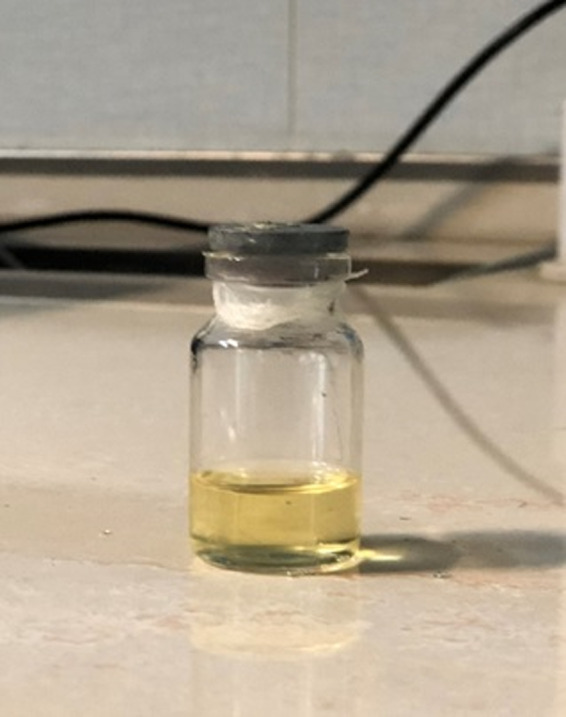
The transparent appearance of optimized nanoemulsion containing flaxseed oil

The turbidity study showed that the optical absorbance at 600 nm wavelength for optimal nanoemulsion sample at the first day and after 11 months of storage at room temperature was 0.08 ± 0.03 and 0.11 ± 0.05, respectively. Turbidity alteration within 11 months of storage is negligible. The turbidity of the nanoemulsion is achieved by the suspended particles. In fact, the turbidity is proportional to the absorption of light by scattered particles of different sizes, which gives a cloudy appearance to the nanoemulsion. Therefore, the nanoemulsion droplets should be smaller than 100 nm to give transparent look (Uluata et al., 2016). The results of visual analysis also testify the transparency of the optimized nanoemulsion (Figure 2). Therefore, the low turbidity and transparent appearance of the optimized flaxseed oil nanoemulsion facilitate its usage for the fortification of transparent foods (Yao et al., 2015).

### 3.3 The amount of omega‐3 content in nanoemulsion containing flaxseed oil

**FIGURE 3:**
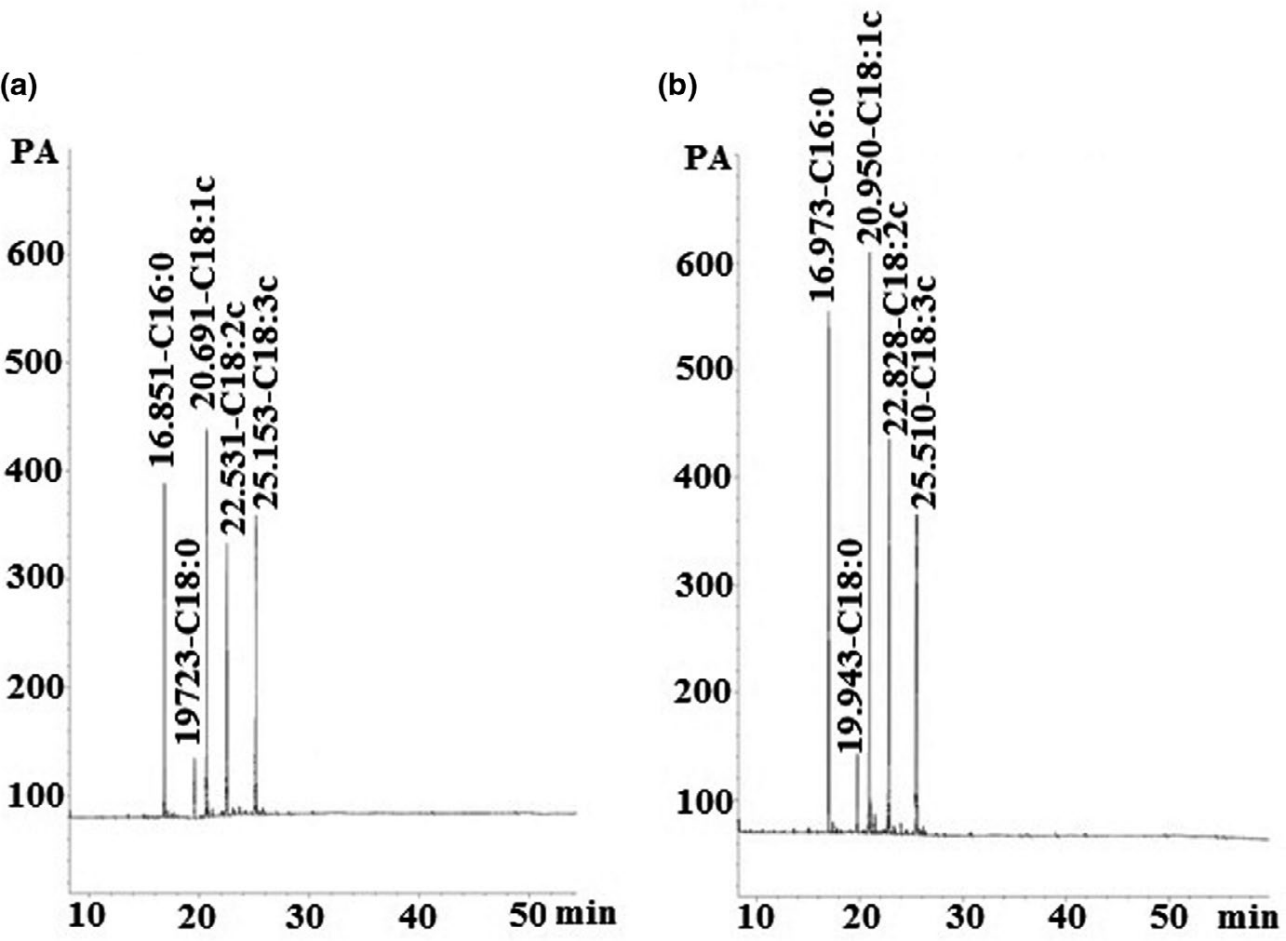
Gas chromatogram graphs of nanoemulsion containing flaxseed oil prepared after (a) 1 day and (b) 11 months

Figure 3a demonstrated the GC analysis of the nanoemulsion containing flaxseed oil. GC quantitative data indicated that the amount of omega‐3 content in nanoemulsion containing flaxseed oil was 27.3%. In order to qualitatively identify the materials with GC device, retention time (RT) is used. By comparing the retention time of the standard sample with the retention time of the unknown sample, the components in the unknown sample can be identified. Quantitative data are usually measured based on the area under the curve (AUC) of each peak. A total of five fatty acid methyl esters were isolated from nanoemulsion containing flaxseed oil. Different fatty acids in flaxseed oil were identified by comparing the RT and AUC of unknown peaks with reference standards. As shown in the diagram, five peaks are related to palmitic acid, stearic acid, oleic acid, linoleic acid, and linolenic acid, respectively, which are in agreement with the analysis of flaxseed oil given in Table 1. The amounts of these fatty acids obtained from chromatographic data were 13.83%, 3.47%, 29.4%, 20%, and 27.3%, respectively. The rest of the small peaks are related to other fatty acids, which consist about 6% of the oil. Figure 3b shows the chromatogram of the same sample after 11 months of storage at room temperature with 19.8% omega‐3 content.

Omega‐3 fatty acid content of flaxseed oil source is about 53.56%, while it decreased to 27.3% in optimal nanoemulsion formulation. It seems that this reduction is due to deficiency of transesterification mechanism. Due to the high molecular weight and low volatility of acyl glycerols, their direct analysis using GC is difficult and measuring fatty acids alone is not feasible by chromatography (except for short‐chain fatty acids such as botanic and pentatonic acid). Therefore, it is better to form fatty acid methyl esters. Transesterification is one of the mechanisms used to make fatty acid methyl esters from fatty esters in fats, including triacylglycerol. Transesterification methods using acidic or alkaline catalysts that can be used to form fatty acid methyl esters in a methanol medium are called trans methylation (internal methylation). Trans methylation is a reversible process and requires an additional amount of methanol to maintain equilibrium, which causes the formation of desirable fatty acid methyl esters. Water can prevent the completion of the reaction, so its presence in the reaction should be limited. Therefore, since the present nanoemulsion contains 51% aqueous phase, the oil phase and the aqueous phase should be separated before performing trans methylation step. The centrifuge and an ultrasonic homogenizer were used for separation, which may affect the amounts of omega‐3 in the oil phase. The non‐quantitative conversion of fatty acids to fatty acid methyl esters, the manipulation of the structure of fatty acids (for example, changes in the position of existing isomers), and the creation of an unmethylated synthetic product may affect the determination of the amount of loaded fatty acids. As a result, the amount reported by the chromatographic method will definitely be less than the real value.

### 3.4 The results of physicochemical characterization of yogurt fortified with omega‐3

The results of measuring pH and acidity at 1 and 21 days after refrigeration (temperature 4℃) are available in Table 4.

**TABLE 4:** Characterization of plain yogurt, yogurt containing bulk oil, and yogurt containing nanoemulsion

Description of test	Unit	Day	Control sample (plain yogurt)	Yogurt fortified with flaxseed oil	Yogurt fortified with nanoemulsion containing flaxseed oil
pH	‐	1	4.08	4.10	4.22
		21	4.08	4.12	4.15
Acidity	Weight percentage	1	1.40	1.43	1.41
		21	1.47	1.43	1.42

Acidity and pH are important for the taste of yogurt. As seen in Table 4, pH and acidity of yogurt fortified with nanoemulsion containing flaxseed oil and yogurt fortified with flaxseed oil were close to the control yogurt. This indicates that addition of nanoemulsion containing flaxseed oil did not affect the post acidification of the yogurt. This was in agreement with studies that indicated out post‐acidification of yogurt is independent of composition and solids content (Bong & Moraru, 2014; Damin et al., 2009).

## 4 CONCLUSION

In this study, an optimized nanoemulsion of flaxseed oil, as a source of omega‐3 fatty acid, was successfully prepared using the low‐energy emulsification method. The droplet size of the nanoemulsion was about 60 nm, which stayed stable and transparent during 11 months at room temperature. The omega‐3 amount of optimized nanoemulsion was about 27.3%. Both nanoemulsion containing flaxseed oil and bulk flaxseed oil were used to fortify yogurt. In terms of physical and chemical properties, yogurt containing nanoemulsions was more similar to simple yogurt. Therefore, fortifying yogurt with nanoemulsions containing flaxseed oil can be considered as a solution to increase the intake of daily requirement of omega‐3.

## CONFLICT OF INTEREST

The authors declare no conflict of interest.

## Data Availability

The data that support the findings of this study are available from the corresponding author upon reasonable request.

## References

[fsn32571-bib-0001] Ajith, T. A. , & Jayakumar, T. G. (2019). Omega‐3 fatty acids in coronary heart disease: Recent updates and future perspectives. Clinical and Experimental Pharmacology and Physiology, 46(1), 11–18. 10.1111/1440-1681.13034 30230571

[fsn32571-bib-0002] Alfaro, L. , Hayes, D. , Boeneke, C. , Xu, Z. , Bankston, D. , Bechtel, P. J. , & Sathivel, S. (2015). Physical properties of a frozen yogurt fortified with a nano‐emulsion containing purple rice bran oil. LWT‐Food Science and Technology, 62(2), 1184–1191. 10.1016/j.lwt.2015.01.055

[fsn32571-bib-0003] Amin, N. , & Das, B. (2019). A review on formulation and characterization of nanoemulsion. International Journal of Current Pharmaceutical Research, 11, 1–5. 10.22159/ijcpr.2019v11i4.34925

[fsn32571-bib-0004] Bong, D. , & Moraru, C. (2014). Use of micellar casein concentrate for Greek‐style yogurt manufacturing: Effects on processing and product properties. Journal of Dairy Science, 97(3), 1259–1269. 10.3168/jds.2013-7488 24440261

[fsn32571-bib-0005] Bush, L. , Stevenson, L. , & Lane, K. E. (2019). The oxidative stability of omega‐3 oil‐in‐water nanoemulsion systems suitable for functional food enrichment: A systematic review of the literature. Critical Reviews in Food Science and Nutrition, 59(7), 1154–1168. 10.1080/10408398.2017.1394268 29058947

[fsn32571-bib-0006] Carpenter, J. , & Saharan, V. K. (2017). Ultrasonic assisted formation and stability of mustard oil in water nanoemulsion: Effect of process parameters and their optimization. Ultrasonics Sonochemistry, 35, 422–430. 10.1016/j.ultsonch.2016.10.021 28340947

[fsn32571-bib-0007] Connor, W. E. (2000). Importance of n− 3 fatty acids in health and disease. The American Journal of Clinical Nutrition, 71(1), 171S–175S. 10.1093/ajcn/71.1.171S 10617967

[fsn32571-bib-0008] Damin, M. , Alcântara, M. , Nunes, A. , & Oliveira, M. (2009). Effects of milk supplementation with skim milk powder, whey protein concentrate and sodium caseinate on acidification kinetics, rheological properties and structure of nonfat stirred yogurt. LWT‐Food Science and Technology, 42(10), 1744–1750. 10.1016/j.lwt.2009.03.019

[fsn32571-bib-0009] Gharehcheshmeh, M. H. , Arianfar, A. , Mahdian, E. , & Naji‐Tabasi, S. (2020). Production and evaluation of sweet almond and sesame oil nanoemulsion and their effects on physico‐chemical, rheological and microbial characteristics of enriched yogurt. Journal of Food Measurement and Characterization, 15, 1–11.

[fsn32571-bib-0010] Ghiasi, Z. , Esmaeli, F. , Aghajani, M. , Ghazi‐Khansari, M. , Faramarzi, M. A. , & Amani, A. (2019). Enhancing analgesic and anti‐inflammatory effects of capsaicin when loaded into olive oil nanoemulsion: An in vivo study. International Journal of Pharmaceutics, 559, 341–347. 10.1016/j.ijpharm.2019.01.043 30710660

[fsn32571-bib-0011] Helland, I. B. , Smith, L. , Saarem, K. , Saugstad, O. D. , & Drevon, C. A. (2003). Maternal supplementation with very‐long‐chain n‐3 fatty acids during pregnancy and lactation augments children’s IQ at 4 years of age. Pediatrics, 111(1), e39–e44. 10.1542/peds.111.1.e39 12509593

[fsn32571-bib-0012] Huang, Q. , Yu, H. , & Ru, Q. (2010). Bioavailability and delivery of nutraceuticals using nanotechnology. Journal of Food Science, 75(1), R50–R57. 10.1111/j.1750-3841.2009.01457.x 20492195

[fsn32571-bib-0013] Kentish, S. , Wooster, T. , Ashokkumar, M. , Balachandran, S. , Mawson, R. , & Simons, L. (2008). The use of ultrasonics for nanoemulsion preparation. Innovative Food Science & Emerging Technologies, 9(2), 170–175. 10.1016/j.ifset.2007.07.005

[fsn32571-bib-0014] Klinkesorn, U. , Sophanodora, P. , Chinachoti, P. , McClements, D. J. , & Decker, E. A. (2005). Stability of spray‐dried tuna oil emulsions encapsulated with two‐layered interfacial membranes. Journal of Agricultural and Food Chemistry, 53(21), 8365–8371. 10.1021/jf050761r 16218689

[fsn32571-bib-0015] Kolanowski, W. , & Berger, S. (1999). Possibilities of fish oil application for food products enrichment with omega‐3 PUFA. International Journal of Food Sciences and Nutrition, 50(1), 39–49. 10.1080/096374899101409 10435119

[fsn32571-bib-0016] Qin, Y. , Nyheim, H. , Haram, E. M. , Moritz, J. M. , & Hustvedt, S. O. (2017). A novel self‐micro‐emulsifying delivery system (SMEDS) formulation significantly improves the fasting absorption of EPA and DHA from a single dose of an omega‐3 ethyl ester concentrate. Lipids in Health and Disease, 16(1), 1–11. 10.1186/s12944-017-0589-0 29037249 PMC5644165

[fsn32571-bib-0017] Ruxton, C. , Reed, S. , Simpson, M. , & Millington, K. (2007). The health benefits of omega‐3 polyunsaturated fatty acids: A review of the evidence. Journal of Human Nutrition and Dietetics, 20(3), 275–285. 17539883 10.1111/j.1365-277X.2007.00770.x

[fsn32571-bib-0018] Tadros, T. , Izquierdo, P. , Esquena, J. , & Solans, C. (2004). Formation and stability of nano‐emulsions. Advances in Colloid and Interface Science, 108, 303–318. 10.1016/j.cis.2003.10.023 15072948

[fsn32571-bib-0019] Tur, J. , Bibiloni, M. , Sureda, A. , & Pons, A. (2012). Dietary sources of omega 3 fatty acids: Public health risks and benefits. British Journal of Nutrition, 107(S2), S23–S52. 10.1017/S0007114512001456 22591897

[fsn32571-bib-0020] Uluata, S. , Decker, E. A. , & McClements, D. J. (2016). Optimization of nanoemulsion fabrication using microfluidization: Role of surfactant concentration on formation and stability. Food Biophysics, 11(1), 52–59. 10.1007/s11483-015-9416-1

[fsn32571-bib-0021] von Schacky, C. (2020). Omega‐3 fatty acids in pregnancy—The case for a target omega‐3 index. Nutrients, 12(4), 898. 10.3390/nu12040898 PMC7230742 32224878

[fsn32571-bib-0022] Walker, R. , Decker, E. A. , & McClements, D. J. (2015). Development of food‐grade nanoemulsions and emulsions for delivery of omega‐3 fatty acids: Opportunities and obstacles in the food industry. Food & Function, 6(1), 41–54. 10.1039/C4FO00723A 25384961

[fsn32571-bib-0023] Yao, M. , McClements, D. J. , & Xiao, H. (2015). Improving oral bioavailability of nutraceuticals by engineered nanoparticle‐based delivery systems. Current Opinion in Food Science, 2, 14–19. 10.1016/j.cofs.2014.12.005

[fsn32571-bib-0024] Zhong, J. , Yang, R. , Cao, X. , Liu, X. , & Qin, X. (2018). Improved physicochemical properties of yogurt fortified with fish oil/γ‐oryzanol by nanoemulsion technology. Molecules, 23(1), 56. 10.3390/molecules23010056 PMC6017217 29301277

